# Knowledge, attitudes and practices of young adults towards HIV prevention: an analysis of baseline data from a community-based HIV prevention intervention study in two high HIV burden districts, South Africa

**DOI:** 10.1186/s12889-020-09356-3

**Published:** 2020-08-17

**Authors:** Simukai Shamu, Sikhulile Khupakonke, Thato Farirai, Jean Slabbert, Thato Chidarikire, Geoffrey Guloba, Nkhensani Nkhwashu

**Affiliations:** 1 Foundation for Professional Development, Pretoria, South Africa; 2 University of the Witwatersrand, School of Public Health, Johannesburg, South Africa; 3 National Department of Health, Pretoria, South Africa

**Keywords:** HIV prevention, HIV knowledge, HIV attitudes, HIV practices, Condom use, Young adults, South Africa

## Abstract

**Background:**

With an HIV incidence of 1.00 skewed against women (1.51), adolescents in South Africa are at high HIV risk. This paper assesses young adults’ (18–24 years) knowledge, attitudes and practices regarding HIV prevention in Nkangala and OR Tambo districts.

**Methods:**

A cross-sectional household survey was conducted in two districts in 2017/8. Participants completed computer-assisted self-interviews on HIV knowledge, attitudes, behaviour practices, use of social media and condom use at last sex (proxy for high-risk sex). HIV knowledge was assessed using the South African-adapted UNAIDS scale. Descriptive analyses were conducted and logistic regression models were built to assess factors associated with being knowledgeable of HIV and condom use at last sex.

**Results:**

One thousand nine hundred fifty-five participants were interviewed (90% response rate). Less than half (44.7%) had correct knowledge of HIV prevention and 73% used a condom at last sex. Social media use predicted high HIV knowledge as higher odds were observed among participants using the print media (aOR1.87; 1.34–2.60), WhatsApp (aOR1.55; 1.26–1.90), radio/television (aOR2.75; 1.15–6.55) although social networking sites’ use protected against knowledge acquisition (aOR0.53; 0.34–0.82). Females (aOR0.75; 0.58–0.97) and participants reporting sexual risk were less likely to have HIV knowledge as negative associations were found for having multiple sexual partners in the last 3 months (aOR0.63;0.48–0.82) and ever having sex (aOR0.37;0.23–0.61). Participants who abused drugs (aOR1.40; 1.05–1.88) and had attitudes accepting people living with HIV (aOR2.05; 1.14–3.69) had higher odds of having HIV knowledge. Females (aOR0.70; 0.54–0.91), students (aOR0.52; 0.40–0.66) and participants who abused drugs (aOR0.58; 0.43–0.77) were less likely to report condom use at last sex.

**Conclusions:**

There is a correlation between media use and HIV knowledge, non-condom use and HIV knowledge, and high-risk sexual behaviours and less HIV knowledge. An aggressive community media campaign utilising locally available, preferred and accessible media platforms among young adults is required for behaviour change.

## Background

South Africa has one of the largest HIV burdens in the world with an estimated 7.9 million people living with HIV (PLHIV) making a prevalence rate of about 13% in the entire population, or 20.6% among those aged 15 to 49 years [1]. South Africa contributes one third (33%) of all new infections in East and Southern Africa (UNAIDS 2018) followed by a distant Mozambique with 16% [2]. Nearly 200,000 people in South Africa contracted an HIV infection in 2017. Young women (aged between 15 and 24 years) have the highest HIV incidence of any age or sex cohort, at 2.01% in 2015 and 1.51% in 2016 compared to their male counterparts with 0.49% in 2016 [1, 3, 4]. Young women in their early 20s have a four-fold burden compared to their male peers, with approximately 2000 new HIV infections occurring every week [5]. Despite young women having the highest incidence, it is the young men who do not test for HIV which contributes to challenges to reaching the first 90 (HIV testing) of the UNAIDS’ 90–90-90 goals for South Africa. It is therefore important to develop interventions that target communities with young adults to increase HIV prevention, testing and treatment.

Given the high HIV prevalence and incidence among young adults and the need to meet the 90–90-90 goals, South Africa, through the National Department of Health, launched a community-based HIV counselling and testing (CBCT) programme. To enable the implementation of the programme, a baseline survey to understand the knowledge, attitudes and practices of young adults towards HIV was planned and conducted in two provinces prior to implementing CBCT. The baseline study was aimed at gathering information that would help planning and implementing CBCT interventions and to be used in evaluating the success of the intervention. This paper presents the results of the baseline survey on young adults’ HIV knowledge, attitudes and practices. The paper assesses the level of and factors associated with HIV knowledge and condom use at last sex among young men and women aged 18 to 24 years.

## Methods

### Design and setting

We used baseline cross sectional data from a community-based HIV prevention intervention study on young adult men and women aged between 18 and 24 years. The intervention aimed at increasing HIV knowledge, reducing risky sexual practices and increasing HIV testing and treatment in low income communities in two provinces, Mpumalanga and Eastern Cape. This baseline cross sectional study which was designed to plan interventions, HIV testing and targeting HIV risk people in the community was conducted in two districts - Nkangala in Mpumalanga Province and OR Tambo in the Eastern Cape Province. Data collection took place between October 2017 and January 2018 in low income urban, peri-urban and rural communities that are mostly occupied by black South Africans (97%).

The sampling design used was multi-stage cluster sampling. The methodology for this study has been described elsewhere [6]. The only one district from Mpumalanga province (Nkangala district) was purposively selected while one district from Eastern Cape province (OR Tambo district) was randomly selected from 4 districts for inclusion in the study. The study sample size was calculated as per sample size for prevalence survey with finite population correction. A minimum sample size of 1826 participants was calculated based on a formula that included a precision of 5%, power of 80% and design effect of 1.5. A sample of 29% (22 out of 76) health facilities were selected using simple random selection in the districts. Those facilities that were selected were used as the first stage clusters for the catchment areas in which the study was conducted. The second stage clusters were the catchment areas served by the selected health facilities in the districts. Areas served by facilities were then randomly selected for inclusion in the study. Participants were then consecutively recruited on a gender ratio of 1:1 – for men and women in each district to end up with 1826 participants. Participants were then recruited while using residential maps starting from the first numbered residential house until the sample size was reached in each ward before moving to the next selected ward. Not more than one person per household was interviewed.

### Questionnaire development

We developed a questionnaire with several scales and questions for measuring HIV knowledge, HIV risk and condom use. The questionnaire can be accessed here (10.1371/journal.pone.0217836.s001). Participants’ knowledge of HIV was assessed by asking five questions adopted from the UNAIDS conceptualisation of HIV Knowledge [7] that has since been used a number of times in South Africa’s national surveys [8]. An example of an HIV prevention question read: “To prevent HIV infection, a condom must be used for every round of sex’. HIV knowledge was regarded as being able to answer all the five questions correctly. Four stigma questions were used to assess participant’s attitudes towards people living with HIV by indicating one’s level of agreement with each of the statements using a 4-point Likert scale (from 1 = “strongly disagree”, 2 = “disagree”, 3 = “agree” to 4 = “strongly agree”). An example of a statement used is: “I would stay friends with someone even if I found out that he/she has HIV”. The individual responses for each participant were added and scored. We set the level of positive or accepting attitudes towards people living with HIV (PLWH) at 7 [6]. Being guided by the notion that community attitudes more broadly influence individual attitudes and also given that the study was conducted to enable an understanding of community views ahead of a community-wide programme of HIV testing, we assessed community HIV testing attitudes. These were assessed by asking participants their views about their community perceptions on HIV testing [9] using six questions. Participants indicated their level of agreement with each statement using a 4-point Likert scale with answers ranging from 1 = “strongly disagree” to 4 = “strongly agree” [10]. A typical question read, “People in my community are scared to test for HIV because they think that if they test HIV positive, they can never have a relationship again”. A combined individual’s minimum of 12 scores out of a possible 24 scores in all the questions was interpreted as having positive or accepting attitudes. Participants’ condom use at last sex was asked in the questionnaire to understand participants’ current risk of HIV. Participants were also asked about their history of transactional sex, HIV testing and HIV risk perception. Their use of any prohibited drugs such as tik, cocaine, dagga was asked in the questionnaire.

We also asked participants’ use of the media (radio, television, magazines, newspapers, electronic media, social networking sites). Questions asking participant’s demographic characteristics were added to be able to describe their gender, age, occupation, education, socio-economic characteristics (possession of household goods, income source). The English questionnaire was translated to two local languages mostly spoken by participants in their respective districts - isiNdebele in Nkangala and isiXhosa in OR Tambo. It was then back translated by an independent expert to ascertain clarity and correctness of the translation. Participants selected the language of their choice out of the three languages offered on the tool. The questionnaire was set into a RedCap system for data collection [11, 12].

### Data collection

We trained and engaged male and female local fieldworkers in participant recruitment, enrolment and data collection. The data collection procedures, ethics and instruments were pretested in the communities resulting in only minor language corrections done onto the questionnaire. Data collectors moved door to door recruiting participants. Daily tallying of participants enrolled was done to ensure adequate number of participants were enrolled in terms of gender and cluster as outlined in our minimum required sample size per area. Data collection took place over 3 months from October 2017 until January 2018. Data collected were saved onto a study database for analysis. The ‘Knowledge, attitudes and practices data’ link for the study is presented below the references.

### Data analysis

Data were analysed in Stata 13.0 [13]. We used descriptive analyses for participants’ demographic characteristics, HIV knowledge, HIV risk and condom use. We conducted two multiple regression analyses. The first model assessed factors associated with HIV knowledge and the second model assessed factors associated with condom use at last sex. We selected factors for each model based on the literature on knowledge of HIV and condom use as well as considering variables observed in bivariate analyses. Regarding the latter, Hosmer and Lemeshow’s threshold for variable selection for logistic regression [14] was used and variables with a *p*-value of <.250 in bivariate associations were entered into the respective multiple regression model. Age and socioeconomic status were controlled for in the models. The models were built using a stepwise regression approach and the outputs were presented as adjusted Prevalence Odd Ratios (aORs). Our analysis took into account the clustering of the sample.

### Ethics

The study received Ethics approval from the Foundation for Professional Development Research Ethics Committee (6/2017). All participants interviewed provided written informed consent. Participants did not receive an incentive for participation.

## Results

A total of 1955 (88.8% response rate) participants enrolled into the study of which 971 (91.7%) were enrolled in Nkangala district and 871 (88.0%) in OR Tambo district. Half (50.2%) of the participants were female. The median age of the participants was 20 (IQR 19–22) years overall and did not differ by district or gender (*p* > 0.05). More than half the participants (54.9%) were students at either tertiary institutions or secondary school. More women than men reported being unemployed in Nkangala (0.001) and OR Tambo (0.047). The most preferred media channel for communicating HIV and TB messages was the radio (56.4%) followed by the television (52.1%). The print media (magazines 12.7%; newspaper 26.9%) were the least preferred media channel for HIV and TB messages.

### Level of HIV knowledge

Table 1 shows the level of HIV prevention knowledge per individual knowledge item and overall knowledge score by gender. The percentages show the proportion of participants who correctly answered the respective questions while the overall HIV knowledge score shows the proportion of participants who correctly answered all questions. Only 44.7% correctly answered all HIV knowledge questions including correctly rejecting all HIV myths. More males (47.3%) than females (42.1%) were knowledgeable of HIV overall (*p* = 0.027). The question on condom use for risk reduction was the most correctly answered by participants (at least 94%), while the questions on whether AIDS can be cured or not (73.1%) and partner reduction to prevent HIV infection (74.9%) were the least correctly answered by participants. In each case, more males answered correctly than females.

**Table 1:** Knowledge of HIV transmission by gender

HIV Knowledge question	Correct knowledge n/N	Total %	Male %	Female %	***p***-value
Can a person reduce the risk of HIV by having fewer sexual partners?	1409/1881	74.9	77.1	72.7	0.028
Can a person reduce the risk of getting HIV by using a condom every time he/she has sex?	1798/1902	94.5	95.5	93.6	0.077
Can AIDS be cured?	1380/1887	73.1	77.4	68.9	< 0.0001
Can a healthy–looking person have HIV?	1470/1892	77.7	79.6	75.9	0.054
Can a person get HIV by sharing food with someone who is infected with the virus?	1546/1894	81.6	81.8	81.5	0.856
Overall correct knowledge	823/1843	44.7	47.3	42.1	0.027

Significant differences were observed between the two districts as higher knowledge levels were observed in Nkangala than in OR Tambo district (50.5% vs. 38.8%; *p* < 0.0001) overall and in three knowledge domains except for partner reduction and condom use (*p* > 0.05). See Table 2 which shows HIV knowledge levels by district.

**Table 2:** Knowledge of HIV transmission by district

	Total Sample		Nkangala		OR Tambo		
HIV knowledge question	n	%	n	%	n	%	***p***-value
Can a person reduce the risk of HIV by having fewer sexual partners?	1416/1891	74.9	708	74.1	708	75.6	0.451
Can a person reduce the risk of getting HIV by using a condom every time he/she has sex?	1808/1912	94.6	920	93.9	888	93.9	0.187
Can AIDS be cured?	1386/1897	73.1	766	80.6	620	65.5	< 0.0001
Can a healthy–looking person have HIV?	1478/1901	77.8	861	89.6	617	65.6	< 0.0001
Can a person get HIV by sharing food with someone who is infected with the virus?	1553/1904	81.6	836	87.5	717	75.6	< 0.0001
**Overall correct HIV knowledge**	828/1852	44.7	472	50.5	356	38.8	< 0.0001

Table 3 shows demographic, media use and sexual behaviour characteristics of participants by overall HIV knowledge. HIV knowledgeable (high knowledge) participants were less likely to be females (*p* = 0.027), living in substandard housing (*p* = 0.036), living in OR Tambo district (*p* < 0.0001), but were more likely to be living in households with at least five basic commodities (*p* < 0.0001) and married (*p* = 0.003). Regarding media use, HIV knowledgeable participants were more likely to be using the radio/television (*p* < 0.0001), print media (*p* < 0.0001), and WhatsApp (*p* < 0.0001) but less likely to be using the internet for social networking (*p* = 0.09). In terms of sexual behaviours, HIV knowledgeable participants were more likely to be those with accepting attitudes towards people living with HIV (*p* < 0.0001), but less likely to have more than three lifetime sexual partners (*p* < 0.001), engage in transactional sex (*p* = 0.019) and have had sexual intercourse (*p* < 0.0001) than their non-HIV knowledgeable counterparts.

**Table 3:** Demographic, media use and sexual behaviour characteristics of participants by HIV knowledge

	HIV knowledge						
	Total		Knowledgeable		Not Knowledgeable		
Demographic characteristics	n	%	n	%	n	%	***p***-value
Age: 21–24 years (vs <21y)	874	47.9	400	49.1	474	46.88	0.338
Gender: Female	935	50.7	394	47.9	541	53.0	0.027
Married, partnered and/or lives with a partner	243	13.2	130	15.8	113	11.1	0.003
Being a student	827	44.9	351	42.5	476	46.9	0.059
Member of a social club	753	41.5	326	39.9	427	42.8	0.215
Receives a social grant	297	16.3	122	14.9	175	17.3	0.164
Living in a substandard house	93	5.1	32	3.9	61	6.0	0.036
Possession of 5+ basic commodities	1142	61.7	632	76.3	510	49.8	< 0.0001
Living in OR Tambo district	917	49.5	356	43.0	561	54.8	< 0.0001
**Media use**							
Uses radio/TV	1752	95.9	808	98.3	944	93.9	< 0.0001
Uses print media	1359	74.5	664	80.9	695	69.3	< 0.0001
Uses WhatsApp	1553	85.9	747	91.5	806	81.3	< 0.0001
Uses internet for social networking sites	1523	83.4	699	85.0	824	82.1	0.09
**Sexual behaviours**							
Ever had sex	474	25.9	153	18.6	321	31.8	< 0.0001
Attitudes towards PLWH	1694	93.1	793	96.4	901	90.4	< 0.0001
Ever used illegal drugs	399	21.8	195	23.7	204	20.2	0.073
Transactional sex ever	266	16.1	102	13.8	164	18.0	0.019
Multiple sexual partners (3+)	477	35.6	198	30.9	279	40.0	0.001
Condom use at last sex	1106	72.6	512	74.2	594	71.3	0.207

### Condom use

We assessed condom use among participants during last sexual intercourse as a preventative measure against HIV infections. We found 27.4% of the participants reporting not using a condom during last sex. A total of 36.7% reported that it was not easy to get a condom if they wanted it. Among the sexually active participants 62.1% reported using a condom at first sex. More women (67.8%) than men (52.7%) reported using a condom (*p* < 0.0001). A total of 83.5% reported that they were able to buy a condom without feeling embarrassed with more males reporting so than women (*p* < 0.0001). The same number reported being confident of putting on a condom correctly with significant differences observed between men and women (87.8% vs 79.3%; *p* < 0.0001) (results not shown on table). Table 4 shows demographic characteristics, media use and sexual behaviours by condom use at last sex. Participants reporting condom use at last sex were less likely to be older (*p* = 0.018), female (*p* = 0.03), students (*p* < 0.0001), but more likely to be belonging to social/community clubs (0.043). Condom users were also more likely to be those with accepting attitudes towards people with HIV but they were less likely to have used illegal drugs, (*p* < 0.0001), engaged in transactional sex (*p* = 0.032) and have multiple sexual partners (*p* = 0.008).

**Table 4:** Demographic, media use and sexual behaviour characteristics of participants by condom use at last sex

	Condom use at last sex						
		Total	Yes		No		
Demographic characteristics	n	%	n	%	n	%	***p***-value
Age: 21–24 years (vs <21y)	810	52.3	567	50.4	243	57.2	0.018
Gender: Female	797	50.9	560	49.2	237	55.4	0.03
Married, partnered and/or lives with a partner	240	15.3	162	14.2	78	18.1	0.053
Being a student	758	48.4	500	43.9	258	60.6	< 0.0001
Member of a social club	648	42.1	489	43.7	159	38.0	0.043
Receives a social grant	246	15.8	166	14.7	80	18.7	0.057
Living in a substandard house	82	5.3	64	5.6	18	4.2	0.273
Possession of 5+ basic commodities	946	60.0	702	61.3	244	56.6	0.09
Living in OR Tambo district	802	50.9	577	50.4	225	52.2	0.521
**Media use**							
Uses radio/TV	1487	95.6	1080	95.6	407	95.5	0.976
Uses print media	1174	75.5	852	75.3	322	76.1	0.726
Uses WhatsApp	1330	86.4	971	86.7	359	85.5	0.534
Uses internet for social networking sites	1323	85.0	967	85.5	356	83.8	0.393
**Sexual behaviours**							
Ever had sex							
Attitudes towards PLWH	1451	93.6	1065	94.5	386	91.0	0.013
Ever used illegal drugs	369	23.6	238	21.0	131	30.5	< 0.0001
Transactional sex ever	257	16.8	173	15.6	84	20.2	0.032
Multiple sexual partners (3+)	474	35.8	326	33.6	148	41.5	0.008
High HIV knowledge	690	45.3	512	46.3	178	42.7	0.207

### Factors associated with HIV knowledge

Table 5 shows the results of the multivariate regression analyses on factors associated with HIV knowledge presented first in unadjusted Odds Ratios (uORs) and then in adjusted Odds Ratios (aORs). The impact of the media on HIV knowledge is strongly evident as people who used the media platforms had higher odds of reporting high HIV knowledge levels compared to those not using the media. Participants who listened to the radio or watched the television compared to those who did not had the highest odds (aOR 2.75 CI 1.15–6.55) of reporting high HIV knowledge levels. Those who used the print media (aOR 1.87) or used WhatsApp (aOR 1.55) also had higher odds of being knowledgeable of HIV than those who reported not using the respective media source. Being female (aOR 0.75), ever having sex (aOR 0.37) and having multiple sexual partners in the last 3 months (aOR 0.63) were associated with lower odds of having high HIV knowledge. Those who used social networking sites were less likely to have higher knowledge scores (aOR 0.53). Having accepting attitudes towards people living with HIV was strongly associated with reporting high HIV knowledge (aOR 2.05).

**Table 5:** Multivariate analysis showing factors associated with knowledge of HIV showing unadjusted and adjusted prevalence odds ratios^a^

Factors	uPOR	95% CI	aPOR	95% CI	
Gender: Female	0.81	0.68–0.98	0.75	0.58–0.97	
Ever used any drugs	1.22	0.98–153	1.40	1.05–1.88	
Ever had sex	0.49	0.39–0.61	0.37	0.23–0.61	
Multiple sexual partners in the last 3mo	0.67	0.54–0.84	0.63	0.48–0.82	
Positive attitudes towards PLWH	2.82	1.85–429	2.05	1.14–3.69	
Listen to the radio/TV	3.73	2.07–6.72	2.75	1.15–6.55	
Use social networking sites	1.24	0.97–1.59	0.53	0.34–0.82	
Use WhatsApp	1.65	1.431.90	1.55	1.26–1.90	
Read print media	2.65	2.14–3.27	1.87	1.34–2.60	

^a^The following variables were included in the model but their relationship with HIV knowledge did not reach statistical level (p>/=0.05): being a student, transactional sex, condom use at last sex, receiving a social grant, knowledge of PrEP, club membership, using radio or television. Data were not weighted

### Factors associated with condom use at last sex

Table 6 shows the results of the multivariate regression analyses on factors associated with condom use at last sex. Participants who were female (aOR 0.70), students (aOR 0.52) or used drugs (aOR 0.58) were less likely to report condom use at last sex. Participants with a high HIV testing knowledge score had 58% chances of using a condom during last sexual intercourse.

**Table 6:** Multivariate analysis results showing factors associated with condom use at last sex showing unadjusted and adjusted prevalence odds ratios ^a^

Factors	uPOR	95% CI	aPOR	95% CI
Gender - Female	0.78	0.62–0.98	0.70	0.54–0.91
Uses drugs	0.60	0.47–0.78	0.58	0.43–0.77
Being a student	0.51	0.41–0.64	0.52	0.40–0.66
High HIV testing knowledge	1.61	1.25–2.09	1.58	1.19–2.08

^a^The following variables were included in the model but did not reach statistical significance (p>/=0.05): having sex in the last 12 months, having knowledge of pre-exposure prophylaxis, engaging in transactional sex, HIV knowledge score. Data were not weighted

## Discussion

The study made four key findings. Firstly and unexpectedly, less than half of the participants were knowledgeable about HIV. Secondly, a high proportion of participants (72%) reported condom use at last sex. Thirdly, media use (television/radio, WhatsApp, print media) factors were found to be key influences of HIV knowledge. Lastly, sexual risk factors including multiple sexual partners and attitudes towards HIV-infected people were associated with HIV knowledge while being knowledgeable of HIV was also associated with condom use at last sex. The following paragraphs discuss these key findings and their implications for community-based counselling and testing for which this study aimed to gather baseline information.

The study unexpectedly found low HIV prevention knowledge (44.7%) among participants. Knowledge levels differed by district and gender. Women had lower levels of knowledge compared to men in both districts and much lower in OR Tambo district. The predominantly rural nature and high poverty levels of participants in the surveyed districts may explain the low levels of HIV knowledge. Several studies have linked HIV knowledge and also infection with poverty and low levels of development [8, 15–18]. The lower knowledge levels in OR Tambo was possibly due to the predominantly rural setting of many clusters surveyed in OR Tambo compared to Nkangala whose greater population is more exposed to urban life in the Capital City, Tshwane. Several communities that we surveyed in Nkangala acted as dormitory towns for Tshwane. More information is needed to assess these geographical differences considering the Ndebele and Xhosa cultures that make up the two communities, Nkangala and OR Tambo respectively. Although we found low knowledge levels among the participants (44.7%), these are higher than found in other studies (27% in Shisana et al. [8] study). While Shisana et al. [8] found that there were significant decreases in knowledge as reflected in their series of surveys since the 2009 survey [19], our finding shows an increase instead of a decline if we consider the 2009 Shisana survey as a baseline. By any standards, these knowledge levels are unacceptably low considering that participants were asked basic HIV questions which ordinarily are expected to be known, three decades since the first HIV case was discovered, also given the sexual and HIV education in schools and in the media. This calls for an aggressive strategy for community-based HIV prevention marketing and testing [4] to address the persisting knowledge gaps.

Another unexpected finding, in a positive direction though, was the high rate of condom use at last sex (72.6%) when compared to what other studies found. Our figure is double the one found in a South African 2012 national survey [8] which found only 36.2% of the participants reporting condom use at last sex [20]. The difference most probably signals behaviour change towards increased condom use in the population. We cannot speculate that there was interviewer bias since our interviews were self-administered on a tablet which gave the participant freedom and a good environment to answer the questions, free from interviewer bias. We understand that our survey enrolled a specific population, 18 to 24 year olds who were mostly single and not living with their partners, while Shisana eta al [8] enrolled 15–49 year olds who are mostly married and living together, factors that predispose people to not use condoms. Studies have shown that married or cohabiting people do not use condoms at most recent sexual intercourse than those neither married nor not cohabiting [21, 22]

We found positive associations between participants who use the mass media and being knowledgeable of HIV. This association may be a testimony to the power of the media in educating young adults about HIV. Exposure to the media has been effectively found to help positively educate people and change negative HIV behaviours [23–27]. Although some studies found the television only as the most significant medium for TB and HIV prevention messages [8], we found the radio, WhatsApp and print media as equally important in HIV education. While we found the print media to be the least preferred media channel in disseminating HIV/TB messages, it had the strongest association with HIV knowledge after the radio/TV among all the media types. This may be because the print media is linked to high levels of user literacy which may mediate for the relationship between print media and HIV knowledge. Further research is required to understand this relationship. The significant effect of the WhatsApp platform could be because of the accessibility and affordability of internet facilities while the negative association with the internet facility could be that exposure to the internet was mainly for purposes such as entertainment other than educational purposes.

Gender differences in HIV knowledge and condom use were observed in this study. First, contrary to what was found in the national survey that there were no differences between knowledge levels by gender [8], we found gender differences as fewer women were knowledgeable of HIV than men. Secondly, women, compared to men, had less odds of reporting condom use at last sex. This echoes a finding by Peltzer et al. [28]. This may be related to gender inequities and inequalities which are prevalent in South Africa [29, 30]. Very high rates of rape and sexual violence in South Africa which are also explained by gender inequalities have been explained before as major underlying causes of and factors associated with HIV infection [31]. Empowering women and work with boys and men may be a good intervention [32, 33] to increase negotiation of condom use and empowering relations on the part of women for HIV prevention.

The association between mass media and HIV knowledge highlights the importance of mass media in HIV knowledge acquisition. Seventeen studies reviewed in a systematic review on mass media’s effectiveness on HIV outcomes illustrate that mass media positively influences and results in condom use [34]. Johnson et al. attempts a critical explanation of the relationship by arguing that social and behavioural communication programmes including mass media do not directly impact on HIV incidence and prevalence but through the availability of sociocultural norms and commodities such as condoms [9]. We however, did not find an association between mass media and condom use which some have found [21]. Instead, our results show that mass media was associated with HIV knowledge and that HIV knowledge was associated with condom use. Although we did not conduct a causal pathway analysis, we speculate that condom use at last sex could have been influenced by having high HIV knowledge which could have been an effect of exposure to the mass media in the communities. A causal pathway analysis or an intervention study is required to understand the relationship further. The relationship between knowledge of HIV testing and condom use at last sex is encouraging in this era of high HIV prevalence and incidence in South Africa as it informs us to find ways to intervene. We believe that strengthening HIV and AIDS prevention campaigns such as LoveLife, Soul City and Soul Buddyz [28] in the surveyed communities may fast track closing the HIV knowledge, testing and condom use gaps.

The study found that those who were sexually active or engaged in high risk sexual activities (ie people who abused drugs, were sexually active or had multiple sexual partners in the last 3 months) had lower odds of having high HIV knowledge. Our finding reiterates the findings from the Shisana et al. national behaviour survey [8] that key populations at high risk such as high risk drinkers had low levels of HIV knowledge. Such high risk people need to be targeted with HIV prevention messages and HIV testing programmes using specially designed and targeted programmes. We found that high HIV knowledge scores were associated with condom use at last sex and that those who held positive attitudes towards people with HIV were more likely to report being knowledgeable of HIV. This may suggest the power of knowledge in influencing young adults’ behaviours. However, since this was a cross sectional study we cannot conclude on the direction of these relationships which maybe be temporal [8] e.g. being knowledgeable made them to engage in less risky sexual behaviours or vice versa. Longitudinal studies are needed to understand causal relationships of the various associations that we found in the study.

The study has its strengths which include a large sample size overall. That the study was a household survey and that we visited households at least three times to locate the identified eligible participant helped to ensure that we adequately covered the study area giving potential participants adequate opportunity to participate in the study. The study clusters also covered urban, semi urban and rural settings enabling us to recruit participants with characteristics representative of communities and districts where the study was conducted. This helps to make comparisons with data from other household surveys conducted in South Africa. Although some have argued that questions asking one to self-declare intimate behaviours or socially stigmatised behaviours have challenges eliciting accurate data due to reporting bias the key variables used in the study relied on internationally and nationally tested scales and questions such as HIV knowledge and myths [7, 8, 35]. These questions have over the years been used in South Africa and our data is comparable to a greater extent to a series of studies conducted in South Africa. Methodological strengths of the study include a large sample size for adequate power in data analysis. The study also had a very high response rate of 89%, we enrolled equal numbers of men and women of the same age groups. We acknowledge the limitation that weighting was not done in the analysis.

## Conclusions

We found low HIV prevention knowledge levels which were much lower among women, coupled by inadequate but relatively high levels of condom use at last sex. We learnt from this study that young adults who are at high risk of HIV infection also have lower knowledge levels, a relationship that could be bi-directional and needs further analysis in controlled longitudinal studies. We also found that utilising the various media channels to disseminate HIV prevention messages could be a significant contribution towards HIV prevention. Providing community-based HIV testing and counselling services that incorporate behaviour change and HIV prevention communication could be a game changer to doubly meet HIV prevention and treatment goals. Based on our results – low HIV knowledge, high HIV risk and non-condom use at last sex could be simultaneously targeted by providing HIV prevention knowledge and HIV prevention commodities such as condoms and pre-exposure prophylaxis (PrEP). This may empower young adults both with HIV prevention knowledge and power over themselves.

## Supplementary information

**Additional file 1.** Knowledge, attitudes and practices data. This file contains data used for this manuscript.

## Data Availability

The dataset supporting the conclusions of this article is included with the article.

## Abbreviations

aPOR: Adjusted Prevalence Odds ratio
CBCT: Community-based HIV Counselling and Testing
FPD: Foundation for Professional Development
PLWH: People living with HIV
PrEP: Pre-exposure Prophylaxis
UNAIDS: Joint United Nations Programme on HIV and AIDS
uPOR: Unadjusted Prevalence odds ratio

## References

[1] Human Sciences Research Council (HSRC). The Fifth South African National HIV Prevalence, Incidence, Behaviour and Communication Survey, 2017: HIV Impact Assessment Summary Report. Cape Town: HSRC Press; 2018.

[2] UNAIDS (2018). UNAIDS Data 2018.

[3] L., J. Thembisa version 2.5: A model for evaluating the impact of HIV/AIDS in South Africa. (20165).

[4] SANAC (2017). Let our actions count: South Africa’s national strategic plan for HIV,TB and STIs 2017–2022. South African Natl AIDS Counc.

[5] Johnson LF (2016). Prospects for HIV control in South Africa: a model-based analysis. Glob Health Action.

[6] Shamu S, et al. Study on knowledge about associated factors of tuberculosis (TB) and TB/HIV co-infection among young adults in two districts of South Africa. PLoS One. 2019. 10.1371/journal.pone.0217836. 10.1371/journal.pone.0217836 PMC6553726 31170200

[7] Joint United Nations Programme on HIV AIDS (2014). Global Aids Response Progress Reporting 2014: Construction of Core Indicators for monitoring the 2011 United Nations Political Declaration on HIV and AIDS.

[8] Shisana O (2014). South African National HIV Prevalence , Incidence , Behaviour and Communication Survey, 2012.

[9] Johnson S, Kincaid D, ME Figueroa RD, Mahlasela L, Magni S (2013). The Third National HIV Communication Survey.

[10] Jamieson S (2004). Likert scales: how to (ab) use them. Med Educ.

[11] Harris PA (2012). Research Electronic Data capture (REDCap) - planning, collecting and managing data for clinical and translational research. BMC Bioinformatics.

[12] Harris PA, Taylor R, Thielke R, Payne J, Gonzalez N, Conde JG (2009). Research electronic data capture (REDCap)-A metadata-driven methodology and workflow process for providing translational research informatics support. J Biomed Inform.

[13] StataCorp (2013). Stata Statistical Software: Release 13.

[14] Hosmer DW, Lemeshow S. Applied Logistic Regression. New York: Wiley; 2000.

[15] Psaros C, et al. HIV prevention among young women in South Africa: understanding multiple layers of risk. Arch Sex Behav. 2018. 10.1007/s10508-017-1056-8. 10.1007/s10508-017-1056-8 PMC5966340 29134422

[16] Kharsany ABM, et al. Community-based HIV prevalence in KwaZulu-Natal, South Africa: results of a cross-sectional household survey. Lancet HIV. 2018. 10.1016/S2352-3018(18)30104-8. 10.1016/S2352-3018(18)30104-8 PMC7498647 30021700

[17] Kiene SM, Ediau M, Schmarje KA, Kintu M, Tumwesigye NM. Exploring the potential of savings-led economic strengthening HIV interventions among high-risk economically vulnerable fishing communities in Uganda: associations between use of commitment savings, sexual risk behavior, and problematic alcohol use. AIDS Behav. 2019. 10.1007/s10461-019-02475-y. 10.1007/s10461-019-02475-y PMC6765464 30924063

[18] Swann M. Economic strengthening for HIV prevention and risk reduction: a review of the evidence. AIDS Care Psychol Socio Medical Asp AIDS/HIV. 2018. 10.1080/09540121.2018.1479029.

[19] Shisana O, et al. South African national HIV prevalence, incidence, behaviour and communication survey, 2008: a turning tide among teenagers? HSRC Press; 2009.

[20] Zuma K, et al. New insights into HIV epidemic in South Africa: key findings from the national HIV prevalence, incidence and behaviour survey, 2012. African J AIDS Res. 2016. 10.2989/16085906.2016.1153491. 10.2989/16085906.2016.1153491 27002359

[21] Camlin CS, Chimbwete CE. Does knowing someone with AIDS affect condom use? An analysis from South Africa. AIDS Educ Prev. 2003. 10.1521/aeap.15.4.231.23831. 10.1521/aeap.15.4.231.23831 12866835

[22] Hendriksen ES, Pettifor A, Lee SJ, Coates TJ, Rees HV. Predictors of condom use among young adults in South Africa: the reproductive health and HIV research unit national youth survey. Am J Public Health. 2007. 10.2105/AJPH.2006.086009. 10.2105/AJPH.2006.086009 PMC1913066 17538062

[23] Li L, et al. Mass media and HIV/AIDS in China. J Health Commun. 2009. 10.1080/10810730903032994.

[24] Bertrand JT, Anhang R. The effectiveness of mass media in changing HIV/AIDS-related behaviour among young people in developing countries: World Health Organization - Technical Report Series; 2006. 16921921

[25] Jung M, Arya M, Viswanath K. Effect of media use on HIV/AIDS-related knowledge and condom use in sub-Saharan Africa: a cross-sectional study. PLoS One. 2013. 10.1371/journal.pone.0068359. 10.1371/journal.pone.0068359 PMC3709989 23874598

[26] Farr AC, Witte K, Jarato K, Menard T. The effectiveness of media use in health education: evaluation of an HIV/AIDS television campaign in Ethiopia. J Health Commun. 2005. 10.1080/10810730590934244. 10.1080/10810730590934244 16036730

[27] Noar SM, Palmgreen P, Chabot M, Dobransky N, Zimmerman RS. A 10-year systematic review of HIV/AIDS mass communication campaigns: have we made progress? J Health Commun. 2009. 10.1080/10810730802592239. 10.1080/10810730802592239 19180369

[28] Peltzer K, et al. Impact of national HIV and AIDS communication campaigns in South Africa to reduce HIV risk behaviour. Sci World J. 2012. 10.1100/2012/384608. 10.1100/2012/384608 PMC3504395 23213285

[29] Jewkes RK, Levin JB, Penn-Kekana LA (2003). Gender inequalities, intimate partner violence and HIV preventive practices: findings of a south African cross-sectional study. Soc Sci Med.

[30] Jewkes R, Morrell R (2012). Sexuality and the limits of agency among south African teenage women: Theorising femininities and their connections to HIV risk practises. Soc Sci Med.

[31] Shamu S, et al. Prevalence and risk factors for intimate partner violence among grade 8 learners in urban South Africa: baseline analysis from the Skhokho supporting success cluster randomised controlled trial. Int Health. 2016. 10.1093/inthealth/ihv068. 10.1093/inthealth/ihv068 26637828

[32] Jewkes R, Flood M, Lang J (2014). From work with men and boys to changes of social norms and reduction of inequities in gender relations: a conceptual shift in prevention of violence against women and girls. Lancet.

[33] Peacock D, Barker G. Working with men and boys to prevent gender-based violence: principles, lessons learned, and ways forward. Men Masc. 2014. 10.1177/1097184X14558240.

[34] Bertrand JT, O’Reilly K, Denison J, Anhang R, Sweat M. Systematic review of the effectiveness of mass communication programs to change HIV/AIDS-related behaviors in developing countries. Health Educ Res. 2006. 10.1093/her/cyl036. 10.1093/her/cyl036 16847044

[35] UNAIDS. GLOBAL REPORT: UNAIDS report on the global AIDS epidemic 2013: Unaids; 2013. doi: JC2502/1/E.

